# The poly-gamma-glutamate of *Bacillus subtilis* interacts specifically with silver nanoparticles

**DOI:** 10.1371/journal.pone.0197501

**Published:** 2018-05-29

**Authors:** Elise Eymard-Vernain, Yohann Coute, Annie Adrait, Thierry Rabilloud, Géraldine Sarret, Cécile Lelong

**Affiliations:** 1 BIG, LCBM, ProMD, UMR CNRS-CEA-UGA, Grenoble, France; 2 ISTerre, CNRS-UGA, Grenoble, France; 3 BIG, BGE, EDyP, INSERM-CEA-UGA, Grenoble, France; VIT University, INDIA

## Abstract

For many years, silver nanoparticles, as with other antibacterial nanoparticles, have been extensively used in manufactured products. However, their fate in the environment is unclear and raises questions. We studied the fate of silver nanoparticles in the presence of bacteria under growth conditions that are similar to those found naturally in the environment (that is, bacteria in a stationary phase with low nutrient concentrations). We demonstrated that the viability and the metabolism of a gram-positive bacteria, *Bacillus subtilis*, exposed during the stationary phase is unaffected by 1 mg/L of silver nanoparticles. These results can be partly explained by a physical interaction of the poly-gamma-glutamate (PGA) secreted by *Bacillus subtilis* with the silver nanoparticles. The coating of the silver nanoparticles by the secreted PGA likely results in a loss of the bioavailability of nanoparticles and, consequently, a decrease of their biocidal effect.

## Introduction

Nanoparticles are used more and more frequently in industrial processes, food, medicine, or daily personal-care products. At the end of their life cycle, most of the nanoparticle products are discharged into the environment where they could potentially impact the ecological equilibria [1–4]. For example, silver nanoparticles (n-Ag) are present as an antimicrobial agent in a range of consumer products (such as textiles, food packaging, and cosmetics) [5]. They are easily able to leach from the products and, ultimately, end up in the environment. The majority of the released n-Ag are discharged into sewer systems. In wastewater treatment plants, 90% of Ag is retained in the sludge. It is now well known that in sewer systems and during wastewater treatment, n-Ag transforms into Ag_2_S [6–10].

The toxicity of n-Ag to living organisms arises both from the released Ag^+^ and from the nanoparticles themselves [11–13], but a clear picture of the mechanisms involved in the toxicity is still missing. Moreover, the experimental conditions (that is, the physicochemical characteristics of the nanoparticles, the composition of the growth medium and the duration of the exposure) are suspected to influence the toxicity of n-Ag [14,15]. Bacteria are one of the first targets of the nanoparticles in the environment. Some studies have already shown that nanoparticles may affect both bacterial physiology and microbial diversity [16]. Alternatively, some resistance to n-Ag has been observed and has been attributed to an interaction between n-Ag and biocompounds present in the surrounding medium or produced by the bacteria (that is, DNA and proteins) [17,18]. Bacteria are also more tolerant of n-Ag when the nanoparticles are applied on a mature bacterial biofilm [4,14]. Under this growth condition, the extracellular matrix produced by the bacteria protects itself by trapping the nanoparticles [19]. Thus, biomolecules excreted by bacteria seem to act as a buffer against the toxicity of n-Ag.

Despite the observations described above, very few studies have investigated the impact of bacteria on the chemicophysical properties of nanoparticles [20]. Our study is focused on the interactions between n-Ag and a model microorganism, *Bacillus subtilis*, that is present both in the gastrointestinal tract and in the rhizosphere. More precisely, our study is interested in the impact of the bioorganic molecules secreted by *Bacillus subtilis* on the nanoparticles.

*Bacillus subtilis* is studied and extensively used for its ability to secrete numerous biopolymers, also called extracellular polymeric substance (EPS). The EPS consists of polysaccharides, proteins, nucleic acids, lipopeptides and poly-gamma-glutamate (PGA) [21]. The EPS composition is complex and depends on the studied strain of bacteria and the growth conditions (for example, a medium that is liquid, solid, rich or minimal). These molecules have various functions, including antibiotic, fungicide and nutrient mobilisation, and are used for many commercial applications. Because of their tertiary structure and chemical properties, some of the components of the EPS (such as nucleic acids or PGA) can react with metals (such as chromium, zinc, arsenate, copper, lead or cadmium) and can be used for bioremediation of contaminated soils [22]. PGA is also extensively studied because of its utilisation in several industrial processes in the food and medical industries [23–25]. Previously, we have shown that two types of nanoparticles, n-ZnO and n-TiO_2_, drastically modify the central metabolism of *Bacillus subtilis* when exposed to these nanoparticles during the exponential growth phase in liquid culture [26]. By contrast, in the present study, the bacteria are exposed to nanoparticles during the stationary phase. In this scenario we show, on one hand, that n-Ag or n-Ag_2_S, have no significant effect on the intracellular physiology of the bacteria; on the other hand, we show that the secreted bioorganic molecules—and, more precisely, the PGA—specifically interact with the nanoparticles.

## Materials and methods

### Nanoparticles suspension

The silver nanoparticles and silver lactate were obtained from sigma: Ag (ref 758329, 5 wt% in ethylene glycol, <100 nm by TEM) and silver lactate (ref 85210, CH3CH(OH)COOAg, molecular weight: 196.94). The n-Ag_2_S was obtained from PVP-n-Ag after sulfidation as described by Levard and collaborators [8]. Briefly, a suspension of 1mM of PVP-n-Ag (pH = 7) was mixed with 1mM Na_2_S in a 0.01M NaNO_3_ electrolyte. After 7 days of reaction, the solutions were centrifuged, washed three times with ultrapure water and dried. The total transformation of Ag^0^ from the PVP-n-Ag to n-Ag_2_S was tested by x-ray diffraction (XRD). The physicochemical characteristics of the nanoparticles have been described by Eymard-Vernain and collaborators [20].

### Bacterial strain and culture media

The *Bacillus subtilis* strain used was the 3610 strain (wild type) (personal gift, Maria Laaberki). The cells were grown in LB medium: 10 g/l tryptone, 5 g/l yeast extract and 5 g/l NaCl. After 21 h of *Bacillus subtilis* growth under shaking in LB medium at 37°C (initial OD_600_ = 0.01), the supernatant was recovered by centrifuging the culture at 15,000 g for 15 min and filtrating with 0.2 μm filters.

### Nanoparticles exposures

In liquid growth conditions: Bacterial cells were grown in LB medium (250 ml) in Erlenmeyer flasks for 16 h with an initial OD_600_ of 0.01. After 16 h of growth, the cell cultures were incubated without or with 1 mg/L of n-Ag, 1 mg/L of n-Ag_2_S or 1.8 mg/L of silver lactate (equal to mg/L of silver) for 5 h while being shaken in 250 mL Erlenmeyer flasks. The pellets and supernatants were separated after centrifugation at 15,000 g for 15min. For the cell-free supernatants, the supernatants were incubated without or with 1 mg/L of n-Ag, 1 mg/L of n-Ag_2_S or 1.8 mg/L of silver lactate for 5 h. The mixture was then centrifuged for 15 min at 15,000 g.

Disk diffusion test: 400 μl of a growth culture (168 *Bacillus subtilis* or 3610 *Bacillus subtilis*) in exponentional phase (OD_600_ ≈ 1.0) were applied uniformly on the surface of a LB agar plate before placing the paper disks (Blotting paper, grade 703 from VWR) of uniform size (5mm) on the plate (five by plate). After 16h of bacterial growth at 30°C, 10 μl of H_2_O, or 10 μl of 10 mg/L, 1 mg/L, 0.1 mg/L or 0.01 mg/L of n-Ag were applied on the paper disks. After 72h at 30°C, the average diameter of the inhibition zone was measured.

All experiments were performed in triplicate (three independent growth cultures) with at least two technical replicates.

### TEM

All samples were obtained after centrifugation at 15,000 g for 30 min. The samples were fixed using high-pressure freezing, freeze substitution and resin embedding, and then prepared as 400 nm sections and deposited on a TEM copper grid. Samples were observed using a Tecnai OSIRIS (FEI) in STEM mode and operated at 200 kV with a probe current close to 0.7 nA.

### High-pressure freezing and freeze substitution

Cells were centrifuged at 4,500 g for 5 min. A pellet volume of 1.4 μl was dispensed on the 200-μm side of a type-A 3-mm gold platelet (Leica Microsystems). It was then covered with the flat side of a type-B 3-mm aluminium platelet (Leica Microsystems) and vitrified by high-pressure freezing using an HPM100 system (Leica Microsystems). Next, the samples underwent a process of freeze-substitution at −90°C for 80 h in acetone supplemented with 1% OsO_4_, and they were then warmed up slowly (1°C/h) to −60°C in an automated freeze-substitution device (AFS2; Leica Microsystems). After 8 to 12 h, the temperature was raised (1°C/h) to −30°C, and the samples were kept at this temperature for another 8 to 12 h. The samples were kept for 1 h at 0°C, brought to 30°C and then rinsed four times in pure acetone. The samples were then infiltrated with gradually increasing concentrations of agar low viscosity resin (LVR; Agar Scientific) in acetone (1:2, 1:1 and 2:1 [vol/vol], and pure) for 2 to 3 h while raising the temperature to 20°C. Pure LVR was added at room temperature. After polymerisation for 24 h at 60°C, 70- to 400-nm sections were obtained using an ultra-microtome UC7 (Leica Microsystems) and an Ultra 35° diamond knife (DiATOME). The sections were collected on formvar–carbon-coated 100-mesh copper grids (for TEM) or on grids with a Si3N4 window (for nanoXRF/nanoXANES). The sections for TEM imaging were post-stained for 10 min with 2% aqueous uranyl acetate, rinsed, and incubated for 5 min with lead citrate.

### DNA

After 21 h of growth, 8 ml of WT culture were centrifuged at 3,500 g for 20 min. Next, 800 μl of sodium citrate buffer (1.5 M NaCl, 0.1 M sodium citrate) was added to the cell-free supernatant and incubated at 37°C for 30 min. After 30 min of incubation, 6.5 ml of NaCl was added and samples were centrifuged at 3,500 g for 10 min. Then supernatant was precipitated by the addition of one volume of ethanol and centrifuged at 15,000 g for 10 min. Finally, the sample was dried and resuspended in 50 μl of H_2_O.

### DNA separation

Before separation on 1% agarose gels in TBE buffer, 2 μl of loading buffer in 10 μl of DNA sample was added. To visualise the DNA, SYBR green was added to the gel and ultraviolet revelation was carried out.

### PGA extraction

After 5 h of incubation, 12 ml of each sample were centrifuged at 3,500 g for 20 min at room temperature. The supernatant was precipitated by the addition of three volumes of cold ethanol, followed by the cooling of the supernatant at −20°C for at least 12 h. After the 12 h incubation, the samples were centrifuged at 10,000 g for 10 min, and the PGA pellets were resuspended in 200μL of 300 mM NaCl. All experiments were performed in triplicate (three independent growth cultures) with at least two technical replicates.

### PGA separation

To begin, 10μL PGA samples were incubated with 5μl of benzonase (25 units/μl) for 30 min at 37°C. Before separation on 0.8% agarose gels during 1 h at 90 mA in TAE buffer, 2 μl of loading buffer (Bromophenol blue 5 mg/L, 50% glycerol and 50% running buffer) was added to the PGA samples. PGA was visualised by staining with 0.5% methylene blue in 3% acetic acid for 30 min and destaining in H_2_O. To verify the absence of DNA, SYBR green was added to the gel and ultraviolet revelation was realised.

### Dot blot assay

Nitrocellulose membrane (Bio-Rad, ref 165–0115) was presoaked in a mixture of Tris (250 mM) and glycine (2 M) for 5 min and assembled onto a dot-blot apparatus (Bio-Rad). Next, 50 μl of PGA was extracted, as described previously; the PGA was added to appropriate wells; it was allowed to filter by gravity for 10 min; and it was followed by vacuum filtration for 5 min to remove all liquid from the wells. The membrane was removed and treated with a blocking solution (1% PVP40 in TBS–Tween) on a rocking shaker for 1 h at room temperature. A 1:2,000 dilution of anti-polyglutamate antibody (from Coger, ref: AG—25B-0030-C050) was added to the membrane as a primary Ab, and it was incubated for 1 h at room temperature. After three washes in TBS–Tween–1% PVP40, the membrane was incubated for 1 h with 1:5,000 secondary antibody, anti-rabbit IgG-HRP (Sigma, A0545). After three washes in PBS–Tween, the dot blot was developed using the Luminata Forte Western HRP substrate (from Merck millipore, ref: WBLUF0500). The specificity of the primary antibody (anti-polyglutamate chain) was checked on commercial PGA samples, PGA extracted from *Bacillus subtilis* supernatant culture and on an *Escherichia coli* extract as a negative control (S1 Data).

### Quantification of PGA

After revelation of the intensity of each sample is quantified using ImageJ software then normalised by the control and by the amount of proteins, the PGA samples quantified by Bradford assay. All experiments were performed in triplicate (three independent growth cultures) with at least two technical replicates.

### RT-qPCR

Cell pellets from the different cultures were obtained as described in the section on nanoparticle stress. The cell pellets were lysed and the total RNA was extracted following the supplier’s manual for NucleoSpin® RA II (Machery-Nargel^®^). The RNA preparations were rendered DNA-free using a two-step process. In the first step, water and lithium chloride (LiCl) were added to 60 μl of RNA preparation to create a final volume of 120 μl, with a concentration of 3 M. The samples were chilled for 2 h at -80°C and centrifuged, the pellets were washed twice with 100 μl ethanol and the samples were then centrifuged for 5 min at 13,000 g at 4°C. The supernatant was removed by aspiration and the pellets were dried for 20 min. The pellets were resuspended in 50 μl of H_2_O. In the second step, RNA preparation precipitates with LiCl were incubated with DNase I (RNAse-free) (Sigma-aldrich^®^), following the supplier’s manual. The reverse transcription was realised using the kit manual for Enhanced Avian S RT-PCR of sigma supplier with 1 μg of RNA. Finally, RT-qPCR reactions were carried out in a final volume of 10 μl, with 2 μl of oligonucleotides as primers, 2 μl of cDNA diluted at 1:4 and 5 μl of iTaq universal SYBR Green supermix (BIO-RAD). The RT-qPCR reactions were carried out in automated thermocyclers (Bio-Rad), beginning by 20 sec of heating at 95°C. Then the amplifications were carried out by 39 cycles of incubation at 95°C (30 sec) and 55°C (20 sec). The final step was carried out as an extension to the incubation at 68°C for 5 sec. The RT-qPCR was realised with a fluorescent reporter molecule called SYBR® Green I, which allows the progress of the amplification reaction to be monitored. With each amplification cycle, the fluorescent intensity was measured, and it was proportional to the amplicon concentration. Next, a threshold level of fluorescence was set above the background. The cycle number at which an amplification plot crosses the threshold fluorescence level is called the “Ct”, or threshold cycle. The expression of RNA was normalised by a control condition and by a reference gene, *codY*. All experiments were performed in triplicate (three independent growth cultures) with at least two technical replicates.

### Measurement of the lactate dehydrogenase activity

Cells grown with or without nanoparticle stress were harvested by centrifugation at 13,000 g for 15 min, and the supernatant was taken for detection of LDH release. In 700 μl of supernatant, the following was added prior to analysis: 10 μl of 3 mM phenazine methosulfate, 10 μl of 30 mM of NAD^+^, 5 μl of 42 mM iodonitrotetrazolium, 10 μl of 500 nM lactate and 275 μl of buffer (0.2M HEPES, 2mM MgCl_2_, 2% Triton). The absorbance at 500 nm was measured after 15 min of incubation. A mix without lactate was used as a blank. All experiments were performed in triplicate (three independent growth cultures) with at least two technical replicates.

### Proteomics

The proteomic methods are described in the PRIDE repository PXD007128.

### X-ray absorption spectroscopy

Two samples were studied by Ag K-edge EXAFS spectroscopy: 1mg of PGA was incubated with 500 μg/ml Ag-NPs or with 500 μg/ml Ag lactate during 1h at room temperature. After incubation, the PGA + Ag-NPs suspension was centrifuged and the pellet was freeze dried. The PGA + silver lactate mixture was directly freeze dried. The Ag K-edge reference spectra used for the data analysis included Ag-NPs, Ag lactate in solution (10 mM Ag lactate from Sigma, pH 5) and in solid state after freeze drying, AgNO3 in solution (25 mM AgNO_3_, pH 4.0) and in solid-state, and Ag pectin (polygalacturonic acid) prepared by mixing pectin and AgNO_3_ (500 μg Ag g^-1^ pectin) at pH 6.0, and then freeze drying the solution). Solid state samples were pressed as pellets, and solutions were mixed with 20% glycerol to avoid ice crystal formation during cooling. All spectra were recorded at 15–20°K using a liquid He cryostat on FAME (BM30B) beamline at the European Synchrotron Radiation Facility (ESRF, Grenoble, France) described previously [20]. Data treatment was done with ATHENA from the Demeter software package [27]. After normalization, spectra were analyzed by least-squares linear combination fitting (LCFs) over the *k* range 1–12 Å^-1^ using the standards described above. The fit quality was evaluated by the normalized sum squares residual NSS = Σ [k2 χexp—k2 χ_fit_]^2^ / Σ[k2 χ_exp_]^2^.

## Results

### During the stationary growth phase, exposure to n-Ag has no significant impact on Bacillus subtilis central metabolism

**Fig 1:**
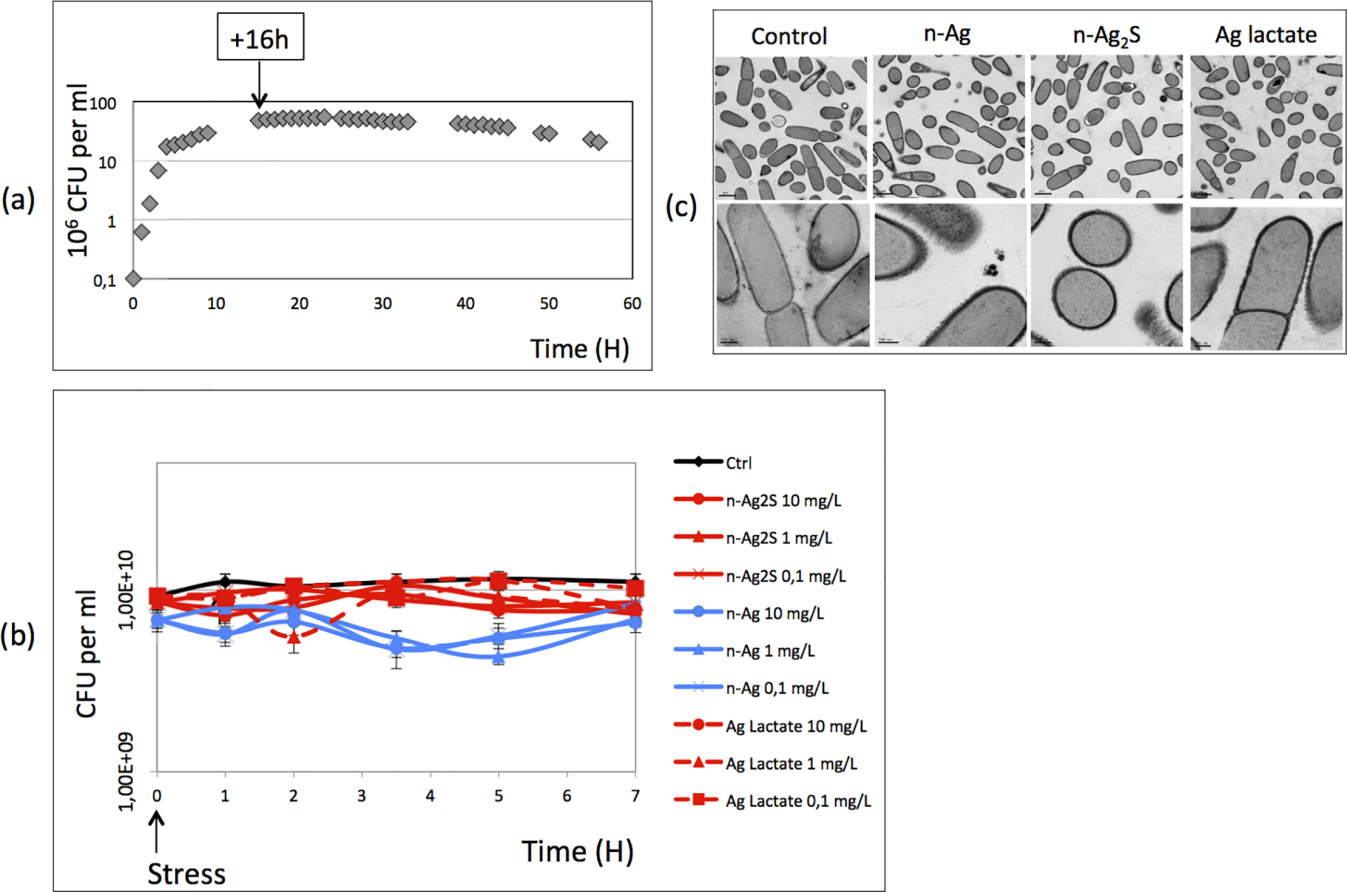
Effect of silver nanoparticles (n-Ag) on *Bacillus subtilis* growth and morphology. (a) Growth curve of *Bacillus subtilis* without Ag: diamond, colony-forming unit. (b) Colony-forming unit of *Bacillus subtilis* exposed to different concentrations (in mg/L) of n-Ag, n-Ag_2_S and silver lactate after 16 h of growth and during 5 h (c) Morphology of *Bacillus subtilis* after incubation with 1 mg/L of Ag. Scale: 1.μm in the first line, and 200nm for second line.

The lack or low availability of chemically complex nutrients corresponds to the natural growth conditions of bacteria in the environment. To try to get as close as possible to recreating these natural conditions and to be able to study the EPS secreted by the bacteria, the responses of *Bacillus subtilis* to n-Ag were studied during the stationary growth phase (before the cell death). To find the optimal growth conditions during which the bacteria were still alive but during which they were exposed to low levels of nutrients, (that is, the stationary phase), we measured the *Bacillus subtilis* growth curve (OD_600_) in LB medium, and we followed their viability by plating onto LB agar over a 30 h period (Fig 1A). In our experimental conditions, the bacteria reached the stationary growth phase after 10 h and began to die after 30 h (Fig 1A). To test the effects of n-Ag under growth conditions that are as close as possible to environmental growth conditions (that is, a complex biological medium that is low in nutrients), *Bacillus subtilis* cells were exposed, after 16 h of growth in LB medium, to silver (specifically, n-Ag, n-Ag_2_S or silver lactate) at concentrations ranging from 0.1 to 10 mg/L during a 7 h period. The viability was assayed all along the exposition by plating on LB agar.

Regardless of the silver concentration tested and the exposure time, the viability was not significantly affected (Fig 1B). As this test would miss a low mortality of the bacteria, we further confirmed this observation by measuring the activity of an intracellular enzyme, the lactate dehydrogenase (LDH), in the cell free supernatant. After 5 h of exposure, the LDH-specific activity was < 1.9 μmol NADH.min^-1^. μg prot with or without exposition to silver (nanoparticles or lactate). Furthermore, the observations of bacteria by transmission electron microscopy (TEM) showed that cells exposed to 1 mg/L of n-Ag, n-Ag_2_S or silver lactate were intact and comparable to the control, with no sign of lysed cells (Fig 1C).

For all of the following experiments, the *Bacillus subtilis* cells were grown in LB medium for 16 h and then exposed for 5 h to 1 mg/L of silver, n-Ag, n-Ag_2_S or silver lactate. A concentration of 1 mg/L was chosen for all of the following experiments in order to be as close as possible to recreating the environmental conditions [28]. As previously shown, this concentration did not induce cell death. However, silver could alter the cellular metabolism, even for viable cells, as demonstrated previously for zinc oxide [26]. In order to investigate such effects, the intracellular proteome was analysed by mass spectrometry (data are available via ProteomeXchange with identifier PXD007128). Under our experimental conditions, no significant modifications of protein expression were detected after exposure to n-Ag, n-Ag_2_S or silver lactate.

Taken together, the viability, the LDH activity and mass spectrometry data led us to conclude that the *Bacillus subtilis* cells did not appear to be affected by an exposure to 1 mg/L of silver during the stationary phase. In view of this hardly detectable impact of silver on *Bacillus subtilis* metabolism under our experimental conditions, our working hypothesis is that secreted molecules interact with n-Ag and buffer their toxic effects. Indeed, *Bacillus subtilis* is studied and extensively used because of its ability to secrete numerous biopolymers and, more specifically, to secrete soluble biopolymers that present an affinity for silver. DNA and PGA are known to have affinity for metals such as Ag^+^, Cu^2+^, Pb^2+^ or Ni^2+^ due to the presence of phosphoryl or carboxyl groups and to their helical three-dimensional structure [23]. These biopolymers were extracted from the cell culture supernatants of growth cultures that were exposed or not to nanoparticles, as described in the Materials and Methods section, and they are tested for their binding abilities to n-Ag.

### Extracellular DNA does not interact with the silver nanoparticles

The extracellular DNA extracted from the supernatant of cells exposed or not exposed to nanoparticles was quantified by spectrophotometric analysis and gel electrophoresis. No significant modifications to the quantity or the quality (that is, the migration profile on gel electrophoresis), was observed in the presence of silver (data not shown). Thus, under our experimental growth culture conditions, the presence of silver had no detectable impact on the extracellular DNA.

### The silver nanoparticles specifically interacted with the PGA

**Fig 2:**
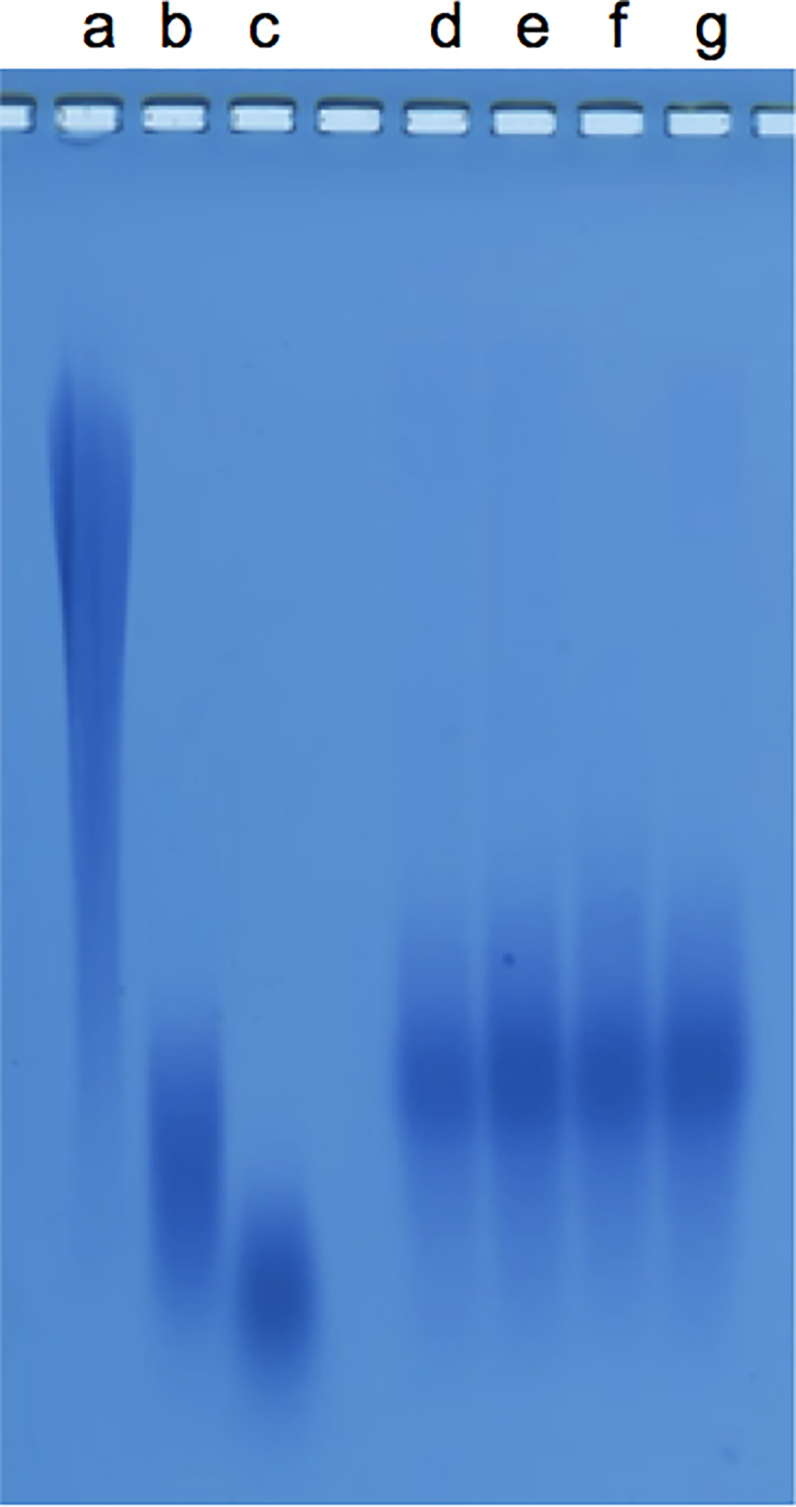
Profile migration of PGA (Poly-g-glutamate) on 0.8% agarose gel electrophoresis. (a) commercial γ-PGA (Sigma Aldrich, G1049); (b) commercial PGA 3–15 kDa (Sigma Aldrich, P4636); (c) commercial PGA 15–50 kDa (Sigma Aldrich, P4886) and PGA extracted from the supernatant of *Bacillus subtilis* growth (d) after no exposure and (e) after exposure to silver nanoparticles (n-Ag), (f) to n-Ag2S or (g) to Ag lactate.

**Fig 3:**
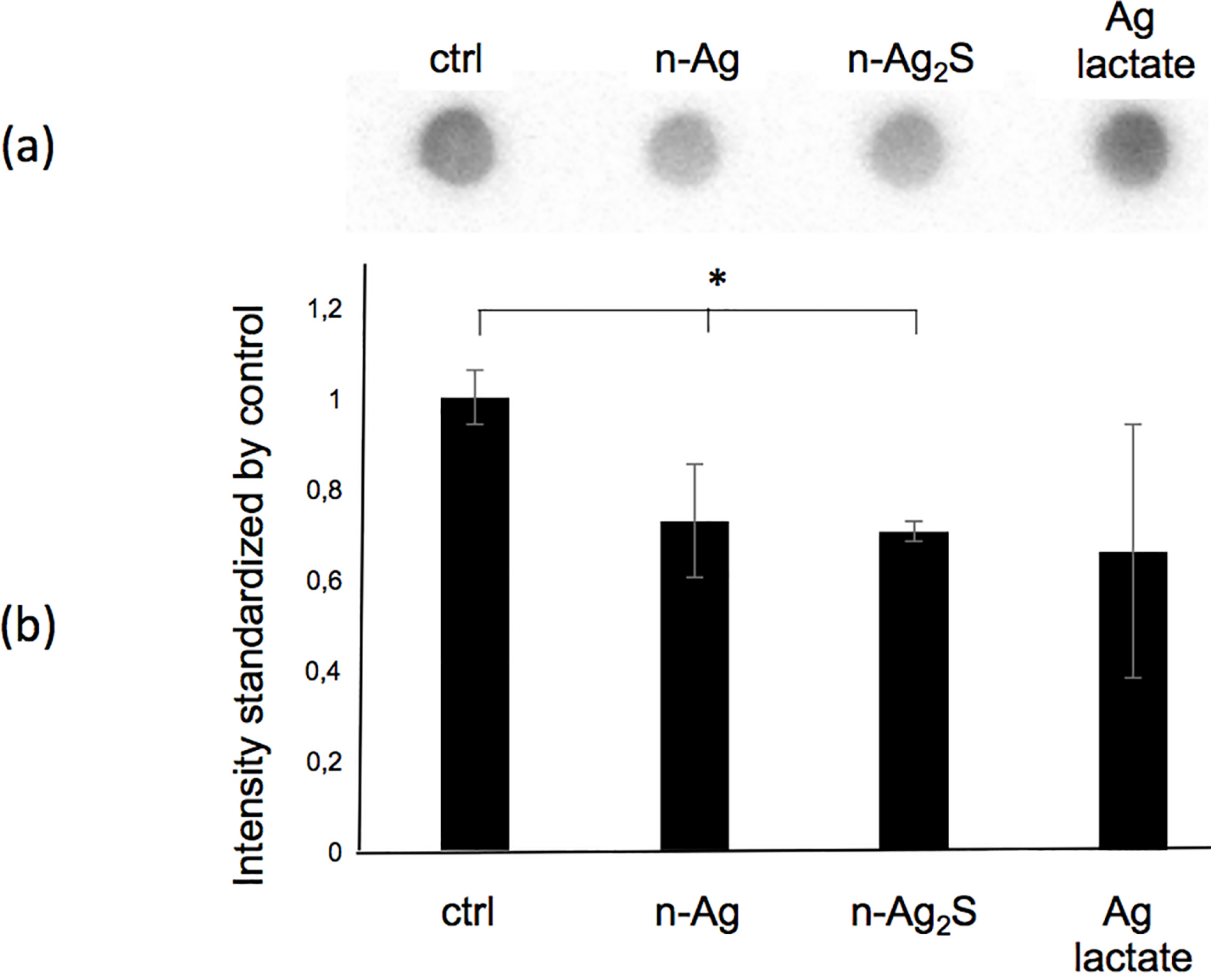
Less PGA is extracted from growth supernatant after exposure of *Bacillus subtilis* to silver nanoparticles (n-Ag). (a) Representative dot blot of the extracted PGA. (b) Quantification of dot blot intensity after stress to *Bacillus subtilis*. The intensities were normalised by the control and the amount of proteins. Error bars ± 1 standard deviation (*n* ≥ 3). Asterisks (*) indicate significant differences vs the control (*p* < 0.05).

**Fig 4:**
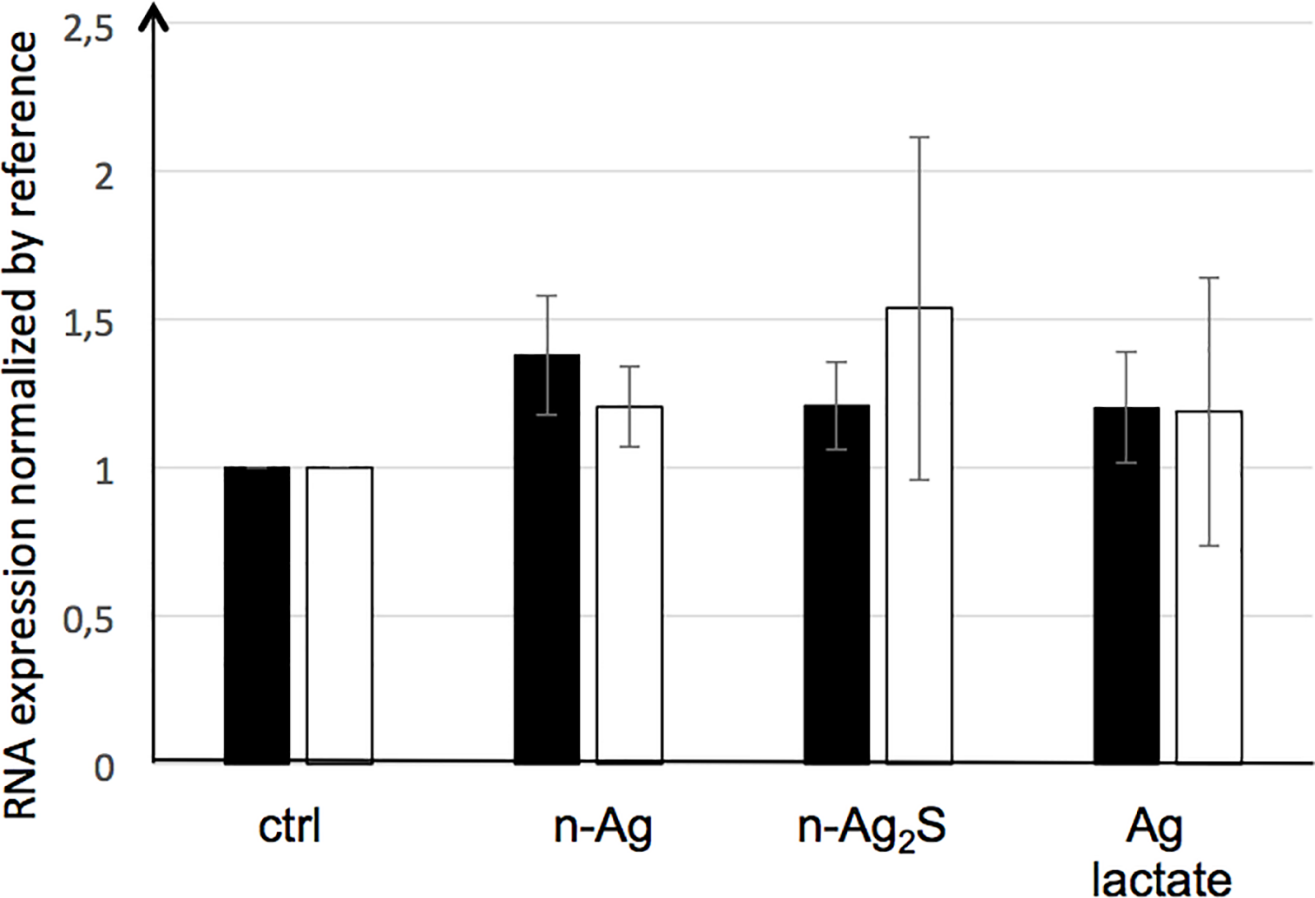
Real-time quantitative polymerase chain reaction (RT-qPCR) of *ggt* and *pgdS* mRNA. The expression of *ggt* (black) and *pgdS* (white) was measured by qRT-PCR on *Bacillus subtilis* exposed to 1 mg/L of silver nanoparticles (n-Ag), 1 mg/L of n-Ag_2_S or 1.8 mg/L of Ag lactate. The RNA expression is described in the Materials and Methods section. Error bars ± 1 standard deviation (*n* ≥ 3).

The presence of silver, n-Ag, n-Ag_2_S or silver lactate had no impact on the quality (i.e. the size) of the secreted PGA, as shown by their agarose gel electrophoresis profiles (Fig 2). Regardless of the experimental growth conditions, all of the secreted PGA displayed the same migration profile, with an average apparent weight of approximately 50 kDa (by comparison with the apparent weight of commercial PGA). This first observation let suppose that the enzymes, pgdS, Ggt or the pgs operon [23,29,30], involved in the PGA metabolism were not affected by the presence of silver in the medium. By contrast, the dot blot analysis, which allows a précised quantification of PGA, showed that the presence of n-Ag or n-Ag_2_S during the stationary growth phase decreased the observed PGA concentrations by about 20%, whereas the silver lactate had no significant effect (Fig 3). The decrease of the extracellular PGA observed in the presence of nanoparticles may be the result of two mechanisms: either physical interactions between the PGA and the nanoparticles, or a physiological response of the bacteria to the presence of nanoparticles in the growth culture medium. This physiological response may be the result of an increase in PGA degradation through the activity of the endo-gamma-glutamyl peptidase PgdS or the exo-gamma-glutamyl peptidase Ggt, or a decrease of PGA synthesis through the enzyme coded by the *pgsBCA (capBCA)* [29,30]. None of these enzymes were detected during the proteomic analysis. Thus, we studied the impact of nanoparticles on PGA metabolism at the transcriptional level using real-time quantitative polymerase chain reaction (RT-qPCR). The total mRNA was extracted 15 and 30 min after the addition of nanoparticles to the growth medium, and the expression profile was identical at both times points (data not shown). The *capBCA* operon was not expressed regardless of the growth conditions tested (data not shown). Ggt and pgdS were expressed, but no significant difference was observed when the cells were exposed to nanoparticles or silver lactate compared to the control (Fig 4).

**Fig 5:**
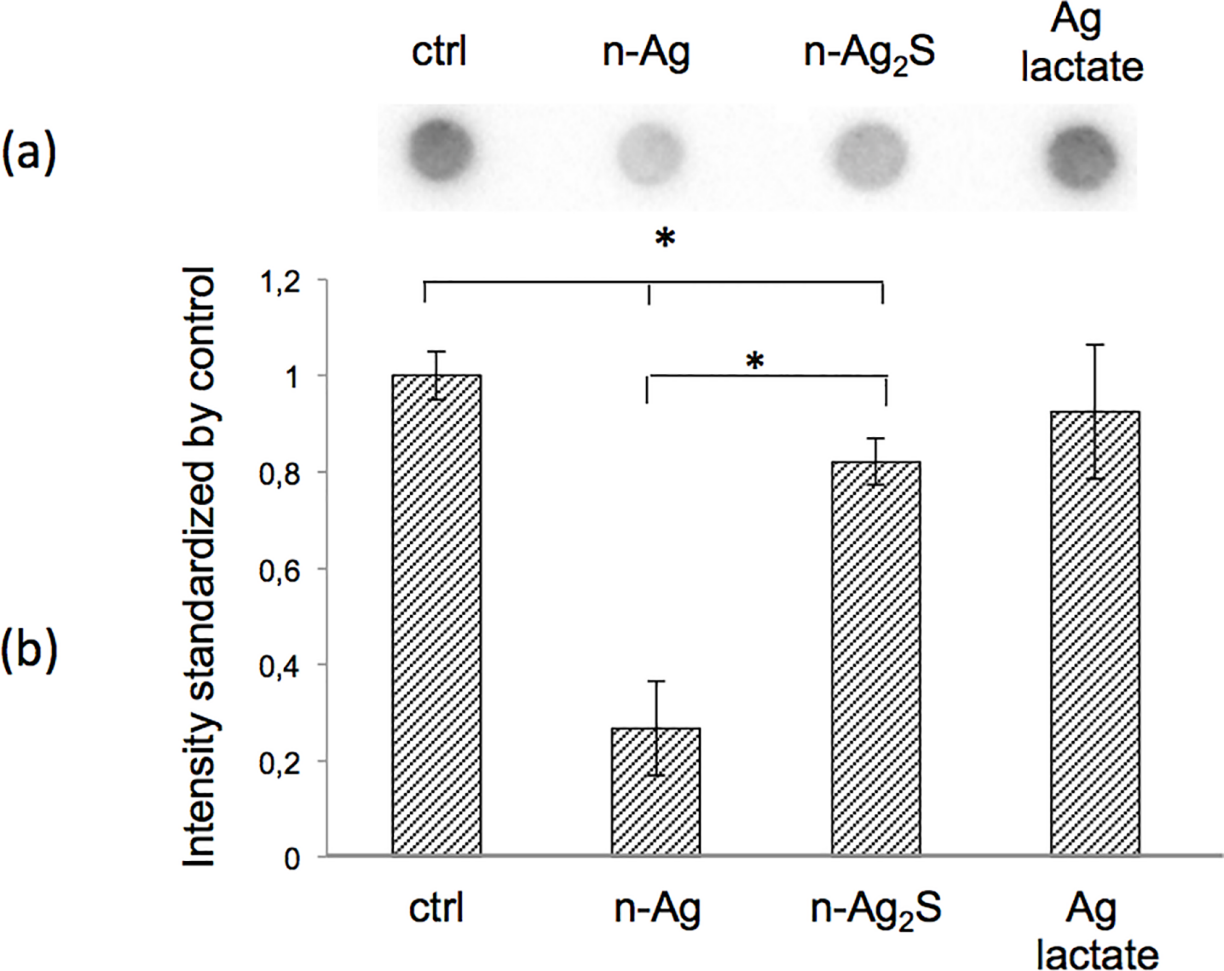
PGA interacts specifically with silver nanoparticles (n-Ag). (a) Representative dot blot of PGA after incubation of cell-free supernatant with n-Ag, n-Ag_2_S or Ag lactate. (b) Quantification of dot blot. The intensity was normalised by the control and the amount of proteins. Error bars ± 1 standard deviation (*n* ≥ 3). Asterisks (*) indicate significant differences vs the control (*p* < 0.05).

**Fig 6:**
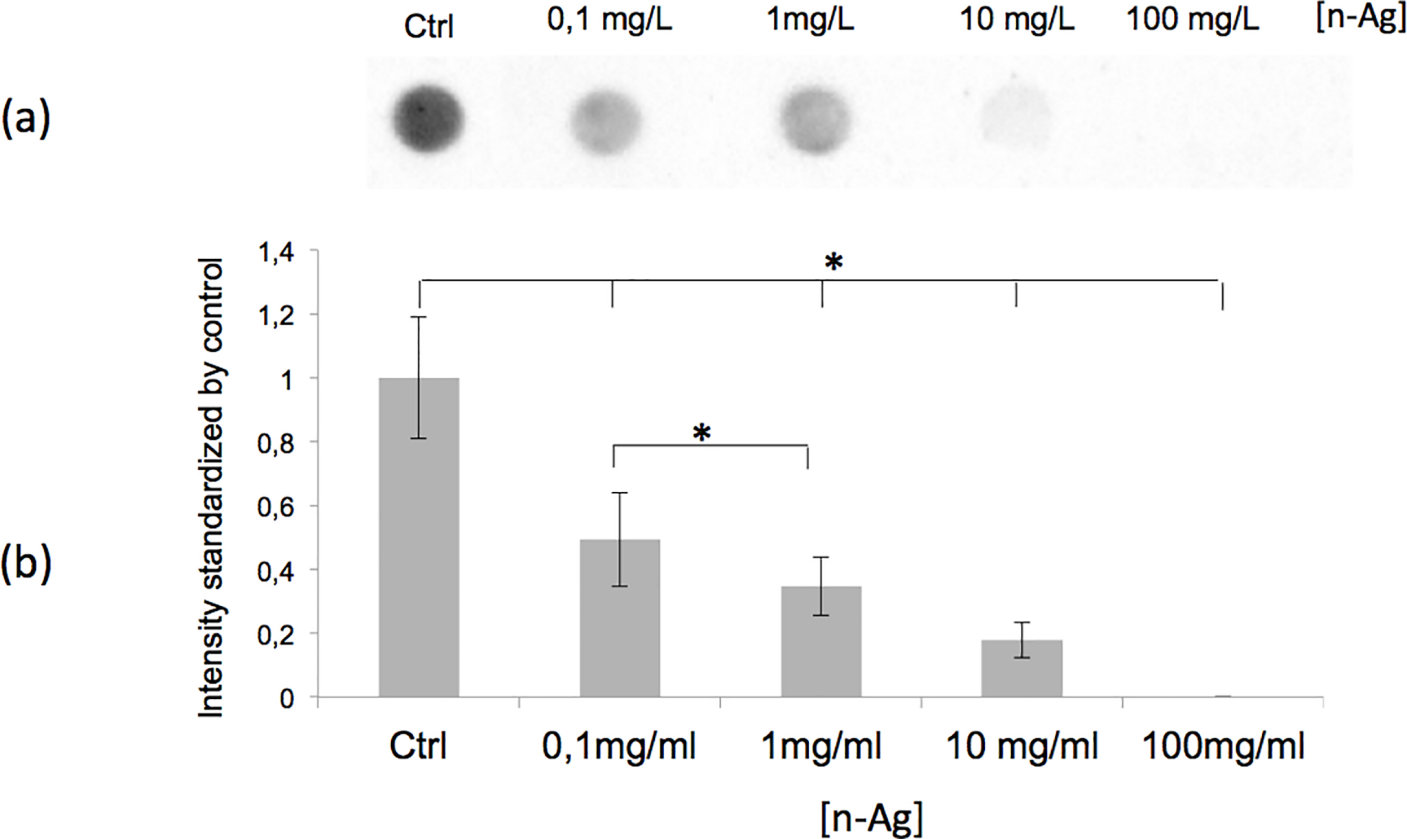
In the cell-free supernatant, the soluble PGA is inversely related to the silver nanoparticle (n-Ag) concentration. (a) Representative dot blot analysis of the soluble PGA in the cell-free supernatant after incubation with n-Ag at different concentrations, ranging from 0 to 100 mg/ml. (b) Quantification of dot blot intensity normalised by the control and the amount of proteins. Error bars ± 1 standard deviation (*n* ≥ 3). Asterisks (*) indicate significant differences (*p* < 0.05).

The results of our experiments allowed us to conclude that under our experimental conditions, PGA metabolism was not affected by n-Ag. Thus, the decrease in extracellular PGA was probably due to a physical interaction with the nanoparticles. To test this hypothesis, the growth culture supernatant without silver was recovered after 21 h of growth, and it was then cleaned by centrifugation and filtration to remove all of the bacteria cells and possible debris. The nanoparticles, n-Ag or n-Ag_2_S, or silver lactate were added to the cell-free supernatant and incubated for 5 h. After centrifugation to sediment nanoparticles and precipitated biopolymers, the soluble PGA was recovered in the supernatant and quantified by dot blot (Fig 5). As with the presence of bacteria, the PGA concentration decreased when nanoparticles, n-Ag or n-Ag_2_S, were added to the cell-free supernatant, whereas the silver lactate has no effect. The control using silver lactate showed that the decrease in soluble PGA could be attributed to interactions with nanoparticles and not to silver ion-induced precipitation. In the absence of bacteria, 20% and 80% of PGA disappeared after the addition of n-Ag_2_S and n-Ag, respectively. To confirm the interaction between the PGA and the nanoparticles, nanoparticles concentrations ranging from 0.1 mg/L to 100 mg/L were added to the cell-free supernatant (Fig 6), and the free PGA was quantified as previously described. The free PGA detected after incubation was inversely related to the concentration of the nanoparticles added.

**Fig 7:**
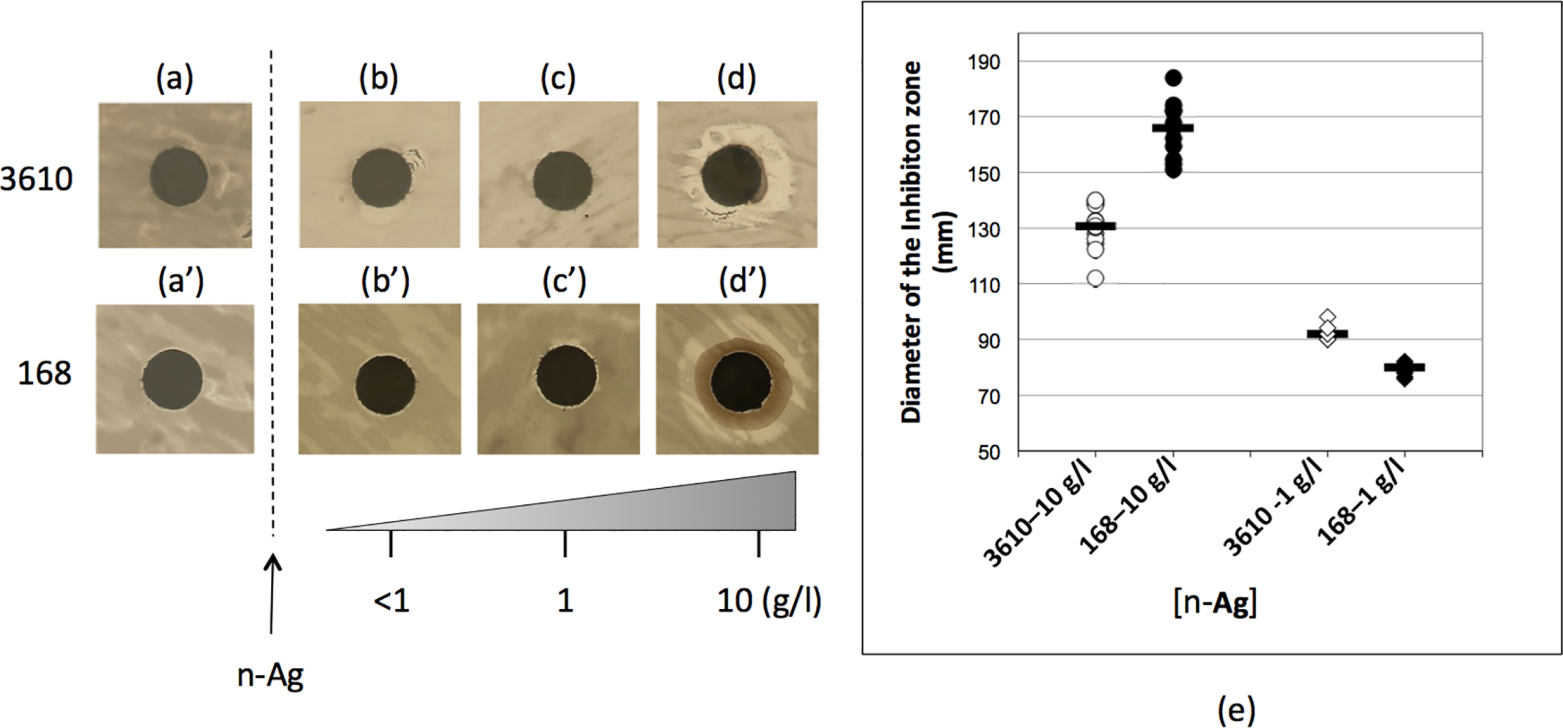
Inhibition zone after addition of n-Ag on *Bacillus subtilis* strains, 3610 and 168, in stationary phase. (a and a’), after 16h of bacterial growth on LB plate without n-Ag, (b-d’) 72 h after addition of n-Ag solutions, (b and b’), 10μl of [n-Ag]≤ 1 mg/l, (c and c’), 10μl of [n-Ag] = 1 mg/l and (d and d’), 10μl of [n-Ag] = 10 mg/l, (e) graph plot of the measured diameter of the inhibition zone after addition of, circle, [n-Ag] = 10 mg/l, diamond, [n-Ag] = 1 mg/l, in white, strain 3610 and in black, strain 168. Black line, median.

We have hypothesized that the interaction between the nanoparticles and the PGA could explain the survival of *Bacillus subtilis* 3610 in presence of n-Ag. The strain 3610 is described as the ancestor of the “domesticated” strain 168: both strains are similar on a genomic level, only a small number of mutations explains the observed phenotype differences [31,32]. One of these differences is the inability of the strain 168 to secrete PGA [33–36]. The strain 168 possess all the biosynthetic genes necessary to the production of PGA but they are not transcribed [33] due to several mutations, in the *degU* [35] and the *swrA* genes [34,36]. We have compared the diameter of the inhibition zone due to the presence of n-Ag on the both *Bacillus subtilis* strains 168 and 3610. The n-Ag were applied after 24h of growth on LB agar plates, i.e. when all the plate is covered by bacteria in stationary growth phase (Fig 7A and 7A’). After 72h of incubation at 30°C in presence of n-Ag, we have observed the diffusion of n-Ag solution only on the plates where the strain 168 has grown (Fig 7D and 7D’). Moreover, the diameter of the inhibition zone was significantly higher for the strain 168 compared to the strain 3610 (Fig 7C and 7E). In our conditions, the strain 168, which do not expressed PGA, was less resistant to n-Ag than the strain 3610.

**Fig 8:**
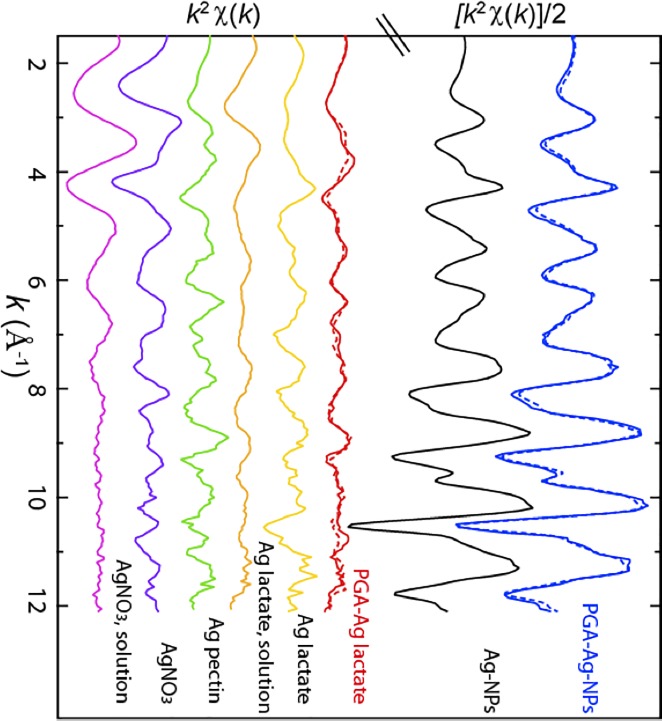
Ag K-edge EXAFS spectra for PGA mixed with Ag lactate and with Ag-NPs, and for various Ag reference compounds. The amplitude of the top two spectra was divided by 2 for better visualization of the other ones. The spectrum for PGA +AgNPs was fitted by 100% AgNPs (dotted line, *NSS* = 0.02), and PGA + Ag lactate was fitted with 38% Ag lactate (solution) + 40%Ag pectin (dotted line, *NSS* = 0.24).

In order to get insights on the type of interaction at the molecular level between PGA and silver in ionic and nanoparticulate form, Ag K-edge EXAFS spectroscopy was used. The spectrum for the silver lactate + PGA mixture differed from the silver lactate alone, and was simulated by about half Ag lactate and half Ag pectin complex (Fig 8). The exact structure of this latter compound is not known, but Ag is probably bound to carboxylates, with some Ag-Ag interactions as described for other polymers [37], visible on the EXAFS spectra by a high frequency contribution (Fig 8). In Ag lactate in solution, Ag is still bound to carboxylate and possibly hydroxyl groups since the spectrum differs from AgNO_3_ in solution (Fig 8), but the interactions are probably weaker since lactic acid is a weak ligand for cations [38]. In the case of the PGA-Ag-NPs sample, the spectrum was identical to the Ag-NPs alone. This is not surprising since EXAFS provides average information on the target element (Ag), and is not sensitive to the minor fraction of Ag atoms present at the surface of the NPs. To conclude, these results suggest that PGA interacts with Ag atoms by binding with carboxyl groups. This is consistent with previous studies on other organic ligands (see the Discussion).

## Discussion

In this study, we have shown using viability assays, TEM observations and proteomic analyses that silver nanoparticles applied on a stationary phase growth culture has no significant effect on *Bacillus subtilis* 3610. Using similar silver nanoparticles concentrations, Gambino and collaborators [39] have described significant effects on protein expression in *Bacillus subtilis*, particularly when considering proteins involved in antioxidant responses. Zhang and collaborators [40] also observed a drastic effects on *Bacillus subtilis* viability; almost 100% lethality after an overnight incubation with 1–2 mg/L of n-Ag. However, in both studies, the nanoparticles were applied at the beginning of the growth phase (that is, during the exponential phase). In contrast, under our experimental conditions, the nanoparticles were applied after 16 h of growth. The cells were in a different physiological state—the stationary phase—during which they had already adapted their metabolism to physiological stress (that is, low levels of nutrients). More generally, during the stationary phase, the bacteria expressed specific proteins to resist to environmental stresses. Our observations are in accordance with the observations that the bacteria are also more resistant to n-Ag when the nanoparticles are applied on a mature bacterial biofilm [4,14]. These results can also be correlated with our previous observations that showed that under these growth culture conditions, no Ag internalisation or adsorption on the cell wall was detected [20].

One way to resist external aggressions is to secrete biomolecules, from enzymes to polymers [21]. One EPS is PGA, which is specifically expressed and secreted during the stationary phase [41–43]. One of the reasons for the survival of *Bacillus subtilis* strain 3610 at the low doses of silver used in this experiment may be the interaction between PGA and the nanoparticles. During all of our experimental conditions, both with the incubation of nanoparticles with living cells and with a cell-free supernatant, we observed a specific interaction between the secreted PGA and the silver nanoparticles, while the extracellular DNA was not affected by the presence of nanoparticles. When the bacteria, in stationary growth phase, were exposed to n-Ag, the survival of the 168 *Bacillus subtilis* strain is lower than those observed for the 3610 strain. Moreover, no diffusion of the n-Ag on the plate (Fig 7D), was observed with the strain 3610, probably due to the interaction of the n-Ag with EPS secreted by the strain 3610. The interaction between EPS and n-Ag could also explain the lower impact of n-Ag on the strain 3610. Two studies have shown that PGA acted as a capping and stabilizing agent for n-Ag [44,45]. In addition, Yu showed that the bactericidal effect of the n-Ag was inversely proportional to the PGA concentration [44]. Similarly, Zhou *et al*. have shown that EPS interacted with n-Ag and alleviated their toxicity to algae [46]. The binding mechanism of EPS with n-Ag involve amine and carboxyl groups. In the case of PGA, the amine group is involved in the polymer formation, so carboxyl groups are the most likely groups bound to Ag.

However, we noticed a significant difference in the percentage of PGA that interacted with the n-Ag under different conditions—20% and 80% when the incubation was performed with living cells and the cell-free supernatant, respectively. No significant difference was detected in the presence of n-Ag_2_S—under both conditions (with cells and cell-free), 20% of the PGA interacted with n-Ag_2_S. These results suggest that the interaction of PGA with the silver nanoparticles is drastically dependent on the speciation of the nanoparticles: the more that the n-Ag are sulfidized, the less that the PGA interact with the nanoparticles.

It is generally considered that n-Ag interacts mostly with thiol-containing molecules that are present in the environment (or with chlorine ions in saline environments). A parallel study of the chemical transformations of n-Ag in the presence of *Bacillus subtilis* and of its secretome demonstrated some sulfidation [20]. The present study showed that biomolecule-bearing carboxyl groups and those that are devoid of thiol groups are also able to interact with n-Ag. Putting the results of the two studies together, it can be concluded that the absence of toxicity of n-Ag may be explained by both the sulfidation process and the interaction between PGA and the nanoparticles, with PGA acting as a capping agent. These results provide evidence of the strong impact of bacteria on the speciation and, therefore, on the bioavailability of n-Ag in the environment.

We have shown here that under our experimental conditions, n-Ag has no impact on *Bacillus subtilis* strain 3610 central metabolism, but it does modify the concentration of free PGA in the medium. The role of PGA in the environment is not completely elucidated, but it is thought to be involved in several biological processes of the bacteria. PGA is also thought to be involved and in the interaction between bacteria and plants, including as a probable carbon source reserve, as a protective factor against dehydration and heavy metals toxicity, in biofilm formation [34]^,^[47], in plant root colonisation [47], as a cryoprotectant that prevents crop from cold-induced damage [48], in the improvement of the capacity of plants to uptake nutrients [49] and as a biocontrol against fusarium root rot [50]. In pathogenic bacilli species, the shell formed by PGA may enhance the virulence of the bacteria by shielding it from the host’s immune surveillance [51], and it may also protect bacteria from phagocytosis and from bacteriophages [52]. In this context, by modifying the availability of PGA, the presence of silver nanoparticles in the soil may impact the metabolism of the rhizosphere and of the plants. Even if bacterial metabolism is unaffected by the nanoparticles, it would be important in the future to study more precisely the fate of the bacterial secretome and its impact on the plants that grow in the vicinity.

We have shown here and in a previous study [20] that bacteria play a crucial role in the fate of n-Ag in a complex biological medium. The presence of *Bacillus subtilis* induces not only the almost total sulfidation of the n-Ag [20], but also influences the interaction of the n-Ag with the secreted PGA. In both cases, the modifications of the nanoparticles likely result in a loss of their bioavailability and, consequently, a decrease in their biocidal effect. But these results raise a new issue—that is, what is the fate of PGA-coated n-Ag in the environment? In a context of controlled wastewater treatment, understanding the interaction between PGA and n-Ag would be of great benefit for the removal of contamination as a result of silver nanoparticles. But in contaminated soil, will PGA-coated n-Ag stay in an inert state or will it migrate into other biological compartments, such as in the plant and then into the gut of cattle during plant ingestion? Several publications describe the role of PGA in the facilitation of drug delivery [23,53–55]. If the PGA-coated n-Ag is ingested, will the PGA shell also promote the nanoparticle delivery into intestinal cells? Considering the biological context, future studies must bear in mind that PGA-coated n-Ag may have a specific biological impact.

## Supporting information

S1 Data Antibodies are specific to PGA. Representative dot blot analysis of commercial PGA (Sigma Aldrich, G1049) and of PGA extracted from *Bacillus subtilis* or *Escherichia coli* supernatant. *Escherichia coli* does no produce any PGA.
(TIFF)
