# Supplementary figures and images for: The poly-gamma-glutamate of Bacillus subtilis interacts specifically with silver nanoparticles
Source: PLoS One. 2018 May 29;13(5):e0197501. doi: 10.1371/journal.pone.0197501 (PMC5973573; doi:10.1371/journal.pone.0197501)

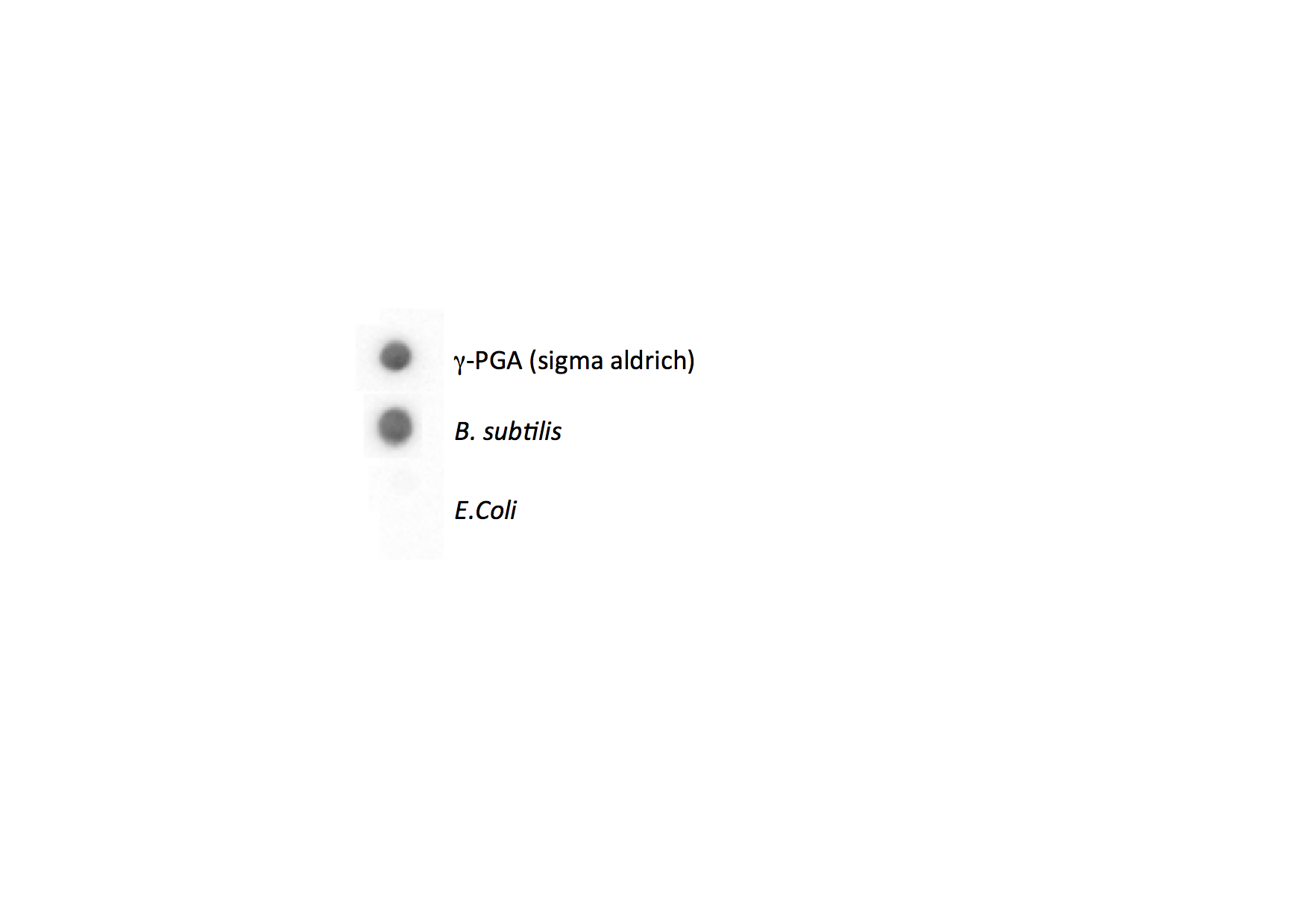

Supplement: S1 Data — Representative dot blot analysis of commercial PGA (Sigma Aldrich, G1049) and of PGA extracted from Bacillus subtilis or Escherichia coli supernatant. Escherichia coli does no produce any PGA. (TIFF) [file pone.0197501.s001.tiff]
